# Genome Sequencing of 15 Clinical *Vibrio* Isolates, Including 13 Non-O1/Non-O139 Serogroup Strains

**DOI:** 10.1128/genomeA.00893-14

**Published:** 2014-09-11

**Authors:** Kimberly A. Bishop-Lilly, Shannon L. Johnson, Kathleen Verratti, Truong Luu, Amy Khiani, Joy Awosika, Vishwesh P. Mokashi, Patrick S. G. Chain, Shanmuga Sozhamannan

**Affiliations:** a Naval Medical Research Center-Frederick, Fort Detrick, Maryland, USA; b Henry M. Jackson Foundation, Bethesda, Maryland, USA; c Los Alamos National Laboratory, Los Alamos, New Mexico, USA

## Abstract

We present draft genome sequences of 15 clinical *Vibrio* isolates of various serogroups. These are valuable data for use in studying *Vibrio cholerae* genetic diversity, epidemic potential, and strain attribution.

## GENOME ANNOUNCEMENT

*Vibrio cholerae* is a Gram-negative bacterium and the etiologic agent of cholera. Cholera is endemic in regions with poor sanitation and can be a significant problem wherever local infrastructure is disrupted due to natural or human-caused disasters. A recent bulletin of the World Health Organization reported that there are an estimated 2.8 million cases and 91,000 deaths annually due to cholera, with the highest burden being in children under the age of 5 years (1).

*V. cholerae* strains can be classified into over 200 serogroups on the basis of differences in the surface-expressed O antigen. Typically, cholera epidemics are associated with specific serogroups, namely O1 and O139, although the recent events in Haiti demonstrate an interesting exception (2). It is currently not known why specific serogroups are associated with epidemic potential even though many other serogroups carry the major virulence factors.

Fifteen clinical isolates of various serogroups from the Sakazaki type strain collection were draft sequenced using the Roche/454 Titanium sequencer (see Table 1). Although all 15 are patient isolates, 13 of them belong to non-O1/non-O139 serogroups.

**TABLE 1:** Listing of strains with serogroups, original country of isolation, and GenBank accession numbers

Strain	Serogroup	Isolation country	Accession no.
NIH41	O1	India	JIDH00000000
5473-62	O31	Philippines	JIDI00000000
1311-69	O35	India	JIDJ00000000
133-73	O48	India	JIDK00000000
1157-74	O53	India	JIDL00000000
981-75	O65	India	JIDM00000000
8-76	O77	India	JIDN00000000
1421-77	O80	India	JMBL00000000
984-81	O89	India	JMBM00000000
571-88	O105	China	JIDO00000000
523-80^*a*^	O115	United States	JIDP00000000
63-93	O139	India	JMBN00000000
234-93	O141	India	JMBO00000000
254-93	O144	India	JMBP00000000
490-93	O155	Thailand	JIDQ00000000

a *Vibrio mimicus*.

Genomic DNA was extracted using the Wizard kit (Promega, Madison, WI). 454 Titanium libraries were prepared and emulsion PCR (emPCR) was performed per the manufacturer’s instructions, prior to multiplexed sequencing and *de novo* assembly. On average, over 500,000 sequencing reads were produced per strain, resulting in genome coverage ranging from 19.4- to 58.4-fold depth.

Preliminary analyses indicate that some of the major *V. cholerae* virulence factors are present in a subset of these genomes. For instance, we identified sequences corresponding to all or a large part of *V. cholerae* pathogenicity island 1 (VPI-1), not only in NIH41 and 63-93 (O1 and O139, respectively), but also in nonepidemic strains 981-75, 1421-77, 571-88, 523-80, and 234-93. We identified sequences with homology to the cholera toxin genes in NIH-41 and 63-93, as expected, as well as in 571-88, 234-93, and 490-93. Finally, we identified sequences corresponding to all or part of VPI-2 in NIH-41 and 981-75. Given that these are draft genomes and in some cases gaps exist within these particular regions, further investigation is required to elucidate whether these virulence factors are functional. These draft genomes have the potential to aid our understanding of virulence and epidemicity in *V. cholerae*. These data contribute to broadening the reference genome collection available for studies aimed at *V. cholerae* genetic diversity and strain attribution.

### Nucleotide sequence accession numbers.

The sequence data were deposited in GenBank under project accession numbers listed in Table 1.
